# Association between the Anatomy of the Mandibular Canal and Facial Types: A Cone-Beam Computed Tomography Analysis

**DOI:** 10.1155/2018/5481383

**Published:** 2018-09-10

**Authors:** Rudyard dos Santos Oliveira, Arlete Maria Gomes Oliveira, José Luiz Cintra Junqueira, Francine Kühl Panzarella

**Affiliations:** Imaging and Oral Radiology, “São Leopoldo Mandic” College, Campus of Campinas, Campinas, SP, Brazil

## Abstract

We evaluated the anatomical variations of the mandibular canal associated with various facial types, age, sex, and side of the face studied. We analyzed 348 hemimandibles in subjects without a history of trauma, lesions in the lower arch, or orthognathic or repair surgery in the posterior mandible. Facial type was determined using the VERT index. The canal path was classified as Type 1 (a large, single structure passing very close to the root tips); Type 2 (a canal passing closest to the mandibular base); and Type 3 (a canal present in the posterior mandibular region, with a lower canal running through the mandibular branch, reaching the anterior region). Bifid canals (type 3) were classified into four categories according to the course and number of mandibular canals. The brachyfacial and mesofacial types presented a Type 1 canal in 95.5% (*n*=166) of subjects, in dolichofacial types, 68.2% (*n*=45) presented a Type 2 canal, while in the mesofacial type, a lower prevalence of the bifid mandibular canal was observed (13.0%, *n*=23) than in the other facial types. The bifid canal showed significant association with facial type only (*p* < 0.05), but no significant association was observed with the anterior loop type (*p* > 0.05). Facial type is significantly associated with the path and morphological variations of the mandibular canal, independently of the side of the face studied, age, and sex.

## 1. Introduction

The mandibular canal is present as a single conduit in most individuals, but may vary with regard to shape (oval, round, or pear-shaped) and whether an accessory canal can be identified (canal bifurcation). Many dentists are unaware of these anatomical variations and thus cannot identify them in radiographic images. Consequently, this can lead to peri- and postsurgical complications, as well as implant planning failure, as it is difficult to predict the exact position of the inferior alveolar nerve [1].

Such anatomical variations, along with operator technique, are a cause of failed inferior alveolar nerve block anesthesia. For instance, an individual who received anesthesia on two separate occasions, but who on both occasions experienced only partial anesthesia of the mandible, was found to have bilateral bifid mandibular canals on radiological examination; this anatomical variation may have affected the results of anesthesia procedures [2].

Thus, identification of bifid mandibular canals may help to prevent complications that can have serious consequences during surgery in the mandibular region. A previous study set out to identify variations of bifid mandibular canals, using computed tomography (CT) scans. From this systematic evaluation of anatomical jaw variations, the authors concluded that bifid mandibular canals are not uncommon; thus, it is important to recognize this anatomical variation prior to performing surgical procedures involving the mandible, and that their presence can be confirmed by three-dimensional imaging techniques [3].

A recent study investigated 603 digital panoramic radiographs of fully dentate patients, with complete root formation. The facial types of these individuals were assessed using cephalometric analysis, based on the VERT index of Ricketts et al. [4], using standard lateral radiographs and sex. The bilateral path of the mandibular canal, assessed on the panoramic radiographs, was classified into three types according to the definition of Nortjé et al. [5]. In Type 1 canals, the mandibular canal was positioned a maximum of 2 mm from the apex of the third molars; in Type 2 canals, the mandibular canal was midway between the root apex of the third molars and the base of the jaw; and in Type 3 canals, the mandibular canal was positioned a maximum of 2 mm from the cortical bone of the jaw base. They showed that there were more Type 2 canals (*p*=0.0012) and fewer Type 1 (*p*=0.0336) canals in female than in male patients, but that there were no associations of canal types with facial types. They therefore concluded that facial type is not associated with the path of the mandibular canal [6].

Nevertheless, the shape, size, and symmetry of other craniofacial structures vary according to the facial type. Verification of different facial types is therefore important for treatment planning in several clinical areas. The facial pattern is a major factor in growth prediction and orthodontic planning. Facial types are described as dolichofacial (vertical growth), mesofacial (balanced growth), and brachyfacial (horizontal growth). There is a positive correlation between the height and average distance from the alveolar process to the upper wall of the mandibular canal. Tall individuals have longer bones than those who are shorter, which can contribute to this correlation [7].

The objective of this study was to evaluate the anatomical variations in the mandibular canal associated with the respective facial types, age, sex, and side of the face studied, using cone-beam computed tomography (CBCT) images and to compare our findings with those of previous studies that used panoramic radiographs.

## 2. Materials and Methods

This study was conducted in accordance with the guidelines established by Resolution 466/12 of the National Council of Ministry of Health and approved by the Research Ethics Committee under Protocol CAAE 58066016.5.0000.5374.

An observational retrospective study was performed using a convenience sample. A total of 174 cases of extended-face cone-beam computed tomography (CBCT) images were analyzed. The scans were all obtained using the same I-Cat® CBCT scanner (Imaging Science, Hatfield, PA, USA) with the following protocol: field-of-view 20 × 16 cm, 0.25 mm voxels, and 20 s, 120 kVp, and 36 mA. The images were assigned to three main groups according to facial type: Group 1, brachyfacial; Group 2, mesofacial; and Group 3, dolichofacial. Patients were divided into two subgroups according to sex (M, male; F, female), and a further subdivision was made according to the side studied: D, right; E, left.

**Figure 1:**
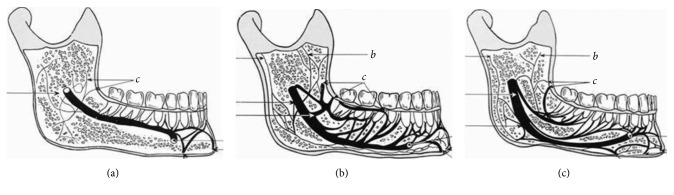
Schematic drawing of the intramandibular course of the inferior alveolar nerve, showing Types 1, 2, and 3 of the mandibular canal, as presented by Carter and Keen [8].

Then, the mandibular canals were classified according to Carter and Keen [8]. In Type 1, the inferior alveolar nerve was a single, large structure located in a bony canal that passed very close to the root tips. In Type 2, the inferior alveolar nerve ran closer to the mandibular base, and the main nerve has small branches that penetrate the root tips. In Type 3, the main branch of the nerve innervates the posterior region of the mandible, while a lower branch traverses the mandible to the anterior region (Figure 1).

**Figure 2:**
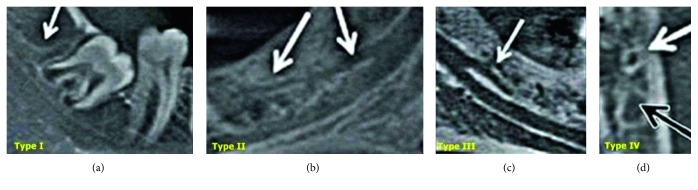
Images presented by Langlais et al. [9] showing different types of bifid mandibular canals. (a) Type I; (b) Type II; (c) Type III; (d) Type IV.

Bifid canals (Type 3) were further classified according to Langlais et al. [9]. Type I canals consist of unilateral or bilateral channels that fork in the mandible, extending to the third molar or adjacent region. Type II channels are also unilateral or bilateral channels that fork in the mandible, but the branches extend along the main channel and rejoin within the mandible body. Type III channels are a combination of the first two categories: the branch of the bifurcated canal extends to the third molar and the surrounding area as in Type I, while the other extends along the main channel, and the branches remain within the mandibular body on the other side, as in Type II. Type IV consists of two channels originating from independent mandibular foramina (Figure 2).

**Figure 3:**
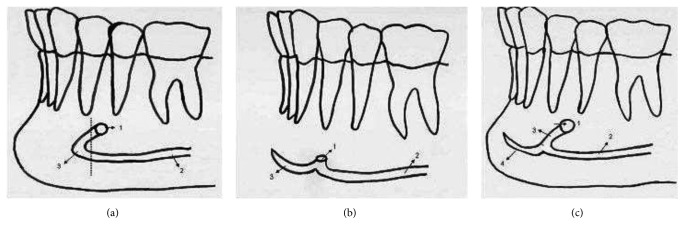
Schematic drawing presented by Greenstein and Tarnow [10]: Type A—schematic drawing illustration: 1, mental foramen outflow; 2, the course of the mandibular canal; 3, anterior loop. Type B—schematic drawing illustration: 1, mental foramen outflow; 2, the course of the mandibular canal; 3, incisive canal without anterior loop. Type C—schematic drawing illustration: 1, mental foramen outflow; 2, the course of the mandibular canal; 3, anterior loop; 4, incisive canal.

In a review by Greenstein and Tarnow [10], the inferior alveolar nerve was described as presenting different morphologies in the mental foramen region. Type A has an anterior loop without any anterior extension (incisive canal). Type B shows the absence of a loop and an anterior extension, while Type C shows the presence of an anterior loop and an anterior extension. To evaluate these variations in the mental foramen region, we followed the schematic shown in Figure 3.

CBCT images were included if they had been obtained due to an indication for orthodontic evaluation and were acquired with the extended face protocol. Images were excluded if they included only the maxillary and mandibular arches. Images that had artifacts that prevented visualizing of the mandibular canal and images of patients with a history of trauma, injuries to the lower teeth, orthognathic surgery, or reconstruction of the posterior mandible were excluded.

To standardize tomographic measurement for classification of facial types and for analysis of the mandibular canal, we performed a pilot study to train the examiner (a radiologist with more than 2 years of experience). We ranked the facial types and mandibular canal types in 30 randomly selected scans and repeated the analyses after 30 days. The results were subjected to analysis of intraexaminer agreement. The intraclass correlation coefficient showed good intraexaminer reliability (kappa = 0.91).

The trained examiner then used Dolphin Imaging Software® version 11.0 (Dolphin Imaging System, Chatsworth, CA, USA) to assess facial type and OnDemand3d® software (CyberMed, Seoul, South Korea) to assess anatomical variations of the mandibular canal in a darkened room.

To determine the facial type, the VERT index of Ricketts et al. [4] was used for cephalometric analysis. Five angular variables were used. (1) The angle of the facial axis (N-BA) (Pt-Gn) is the angle formed by the basion-nasion line with the line of the pterygoid point to the cephalometric gnathion, measured at the posterior angle. The standard angle is 90°, with a standard deviation of +3°, and remains constant with age. (2) The angle of the face or facial depth (E-Or) (N-POG) is the angle formed by the Frankfurt plane and the facial plane. The normal value is 87°, which decreases with age at 0.3° per year. (3) The mandibular plane angle (Go-Me) (Po-Or) is formed by the horizontal Frankfurt plane and the mandibular plane. (4) The height of the lower face angle (Xi-ENA) (X-Pm) is the angle formed by the Xi-ENA and Xi-PM planes. Its standard value is 47° with a standard deviation of +4°, and it remains constant with age. (5) The mandibular arch angle (DC-Xi) (X-Pm) is the angle formed by the body axis and the mandibular condylar axis. Its standard value is 26°, with a 0.5° increase with age for every year of life. This system was used to establish three basic types of facial growth: mesofacial (balanced growth), dolichofacial (predominantly vertical growth), and brachyfacial (predominantly horizontal growth). The cephalometric points of this analysis were obtained using the Dolphin® Imaging program version 11.0 (Dolphin Imaging System). After marking the cephalometric points required for analysis, sagittal reconstruction was performed by overlapping the right and left sides to obtain a full cephalometric tracing. The program allows a close-up view of the area in question, and overlapping points were used to obtained linear and angular values automatically.

**Figure 4:**
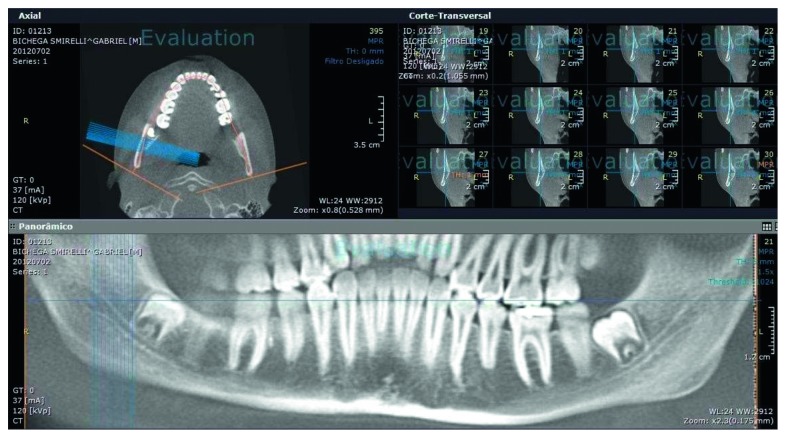
Image from OnDemand3D software, showing the axial slice planes, panoramic reconstruction, and transverse cuts.

Images were processed using OnDemand3d® software. For analysis of CT images, anatomical planes were first corrected using multiplanar reconstruction. Axial images (thickness 0.25 mm) were used to establish a cutting plane along the alveolar ridge of each patient. This was used to obtain transverse slices from the panoramic images. Cross sections of 1.00 mm thickness, with an interslice distance of 1.00 mm, were used for standardization. In panoramic reconstructions, a slice thickness of 5.25 mm was used (Figure 4).

To improve the identification of the mandibular canal, minor changes were made to the bone edge in the cutting plane to correct brightness and contrast, and image filters were applied, as the anatomical structure of this path is not linear and needs to be individualized for each side of the patient. In cases in which bifid canals were detected, buccolingual oblique cuts were made to obtain suitable images.

### 2.1. Statistical Analyses

Data were arranged in absolute and relative frequency distribution tables. Chi-square and Fisher's exact tests were used to analyze the associations of facial type, age, sex, and anatomical variations of the mandibular canal. All analyses were performed using R program (R Core Team, 2015, a language and environment for statistical computing; R Foundation for Statistical Computing, Vienna, Austria (https://www.R-project.org/)). The statistical significance level was set at 5%.

## 3. Results

Of the patients assessed (174 CBCT scans and 348 hemimandibles), 52.9% were female and 47.1% male; 51.1% had a mesofacial type, 29.9% had a brachyfacial type, and 19.0% had a dolichofacial type. No significant associations of facial types with age and sex were observed (*p* > 0.05).

In the analyses of the right side of the face, the location of the mandibular canal was significantly associated (*p* < 0.05) with the facial type. In brachyfacial and mesofacial types, the mandibular canal mostly ran close to the root apexes (63.5% and 58.4% of cases, respectively). In dolichofacial types, the canal mostly ran closest to the base of the jaw, with branches to the root apexes (69.7%) and only 3.0% showed a main canal path running near the root apexes. In mesofacial types, we observed a lower prevalence of bifid mandibular canals than in the other facial types (Table 1).

**Figure 5:**
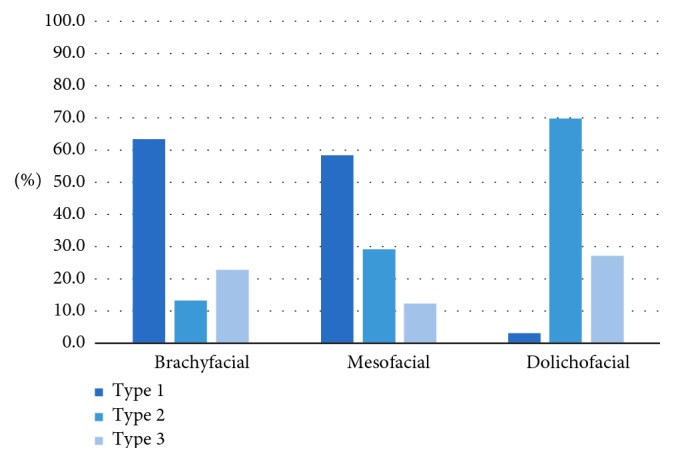
Association between the type of bifurcation in the mandibular canal on the right and the facial type.

The type of bifid canal on the right side was also significantly associated with the facial type (*p* < 0.05) (Figure 5). In the dolichofacial group, there was a higher prevalence of a bifid canal that joins into the base (Type II) than in the other two facial types. Type III bifid canals were only observed in the mesofacial group. Type IV bifid canals were observed only in dolichofacial types.

**Figure 6:**
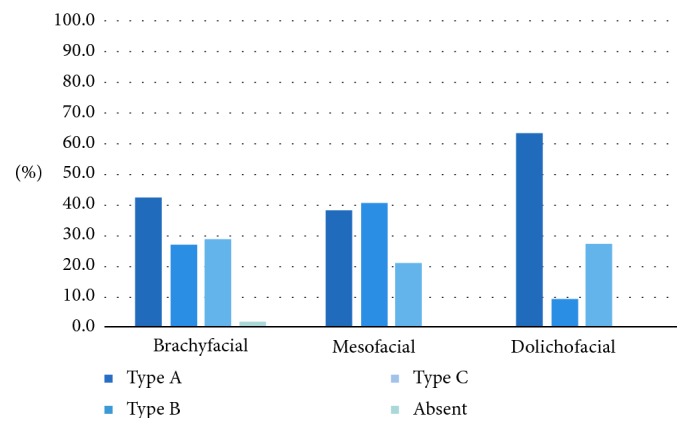
Association between the type of anterior loop on the right and the facial type.

On the right side, the type of loop also showed a significant association with the facial type (*p* < 0.05) (Figure 6). In the dolichofacial group, there was a higher prevalence of an anterior loop (Type A) and a lower prevalence of Type B canals than in the other facial types.

Table 2 presents the results for analysis of the left side of the mandible. The location of the mandibular canal on the left side was also significantly associated with facial type (*p* < 0.05). Again, brachyfacial and mesofacial types mostly presented with a canal running close to the root tips (67.3% and 52.3% of cases, respectively). In dolichofacial types, the main canal path ran closest to the base of the jaw, with branches extending to the root apexes (66.7%), and in only 6.1% did the main canal path run near the root apexes. In mesofacial types, we observed a lower prevalence of bifid mandibular canals than in the other facial types.

**Figure 7:**
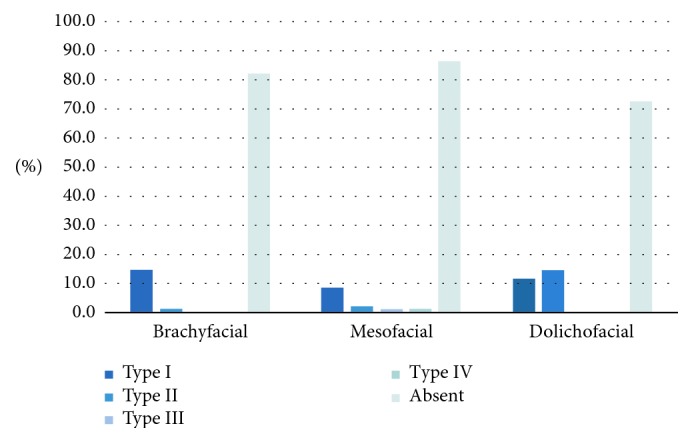
Association between the type of bifid mandibular canal on the left side and the facial type.

**Figure 8:**
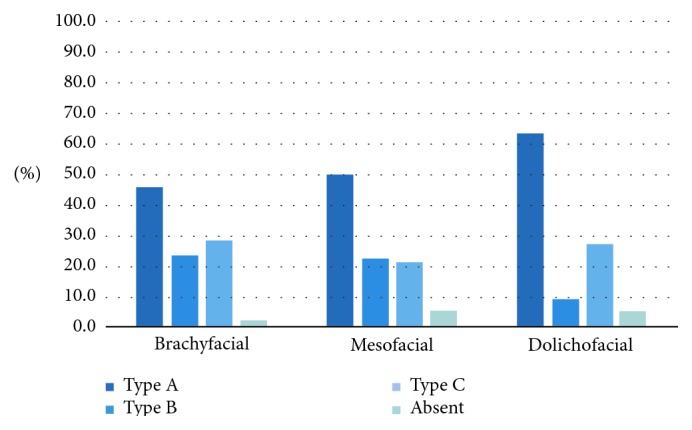
Association between the type of anterior loop on the left side and the facial type.

On the left side, there was no significant association of the type of bifid canal with the facial type (*p* > 0.05; Figure 7). The most prevalent type of bifurcation was the Type I bifurcation, accounting for 11.6%. The only case of two types of bifurcation occurring together was observed in a mesofacial individual. The only case of a bifurcated canal originating from separate foramens was observed in a mesofacial individual. The anterior loop types on the left side were not significantly associated with the facial type (*p* < 0.05; Figure 8), but the most common type encountered was Type A.

Table 3 shows the results of the study, regardless of side. The results of the data overall were similar to that for the sides individually; brachyfacial types and most mesofacial types presented with a canal path running next to the root apexes (65.4% and 55.4% of the studied canals, respectively). In dolichofacial types, canals mostly ran closest to the base of the jaw, with branches extending to the root apexes (68.2% of the canals), and only 4.5% of the canals presented with a course near the root tips. In the mesofacial types, there was a lower prevalence of bifid mandibular canals than in the other facial types.

**Figure 9:**
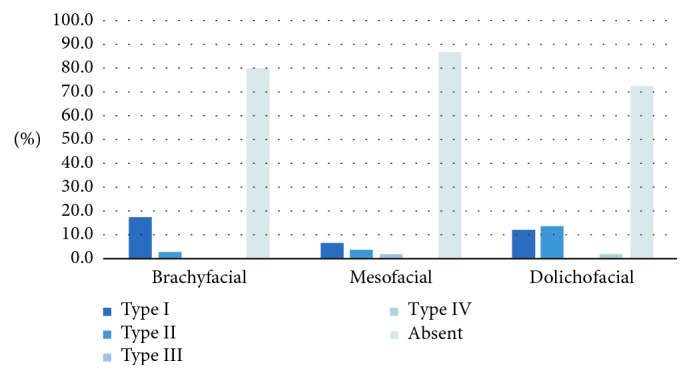
Association between the bifid canal type (regardless of side) and the facial type.

The bifid canal type overall also showed a significant association with the facial type (*p* < 0.05; Figure 9). The dolichofacial group showed a higher prevalence of bifid canals where the bifurcations joined up within the base (Type II) than in the other two facial types. The only cases in which Type III canals were mesofacial types.

**Figure 10:**
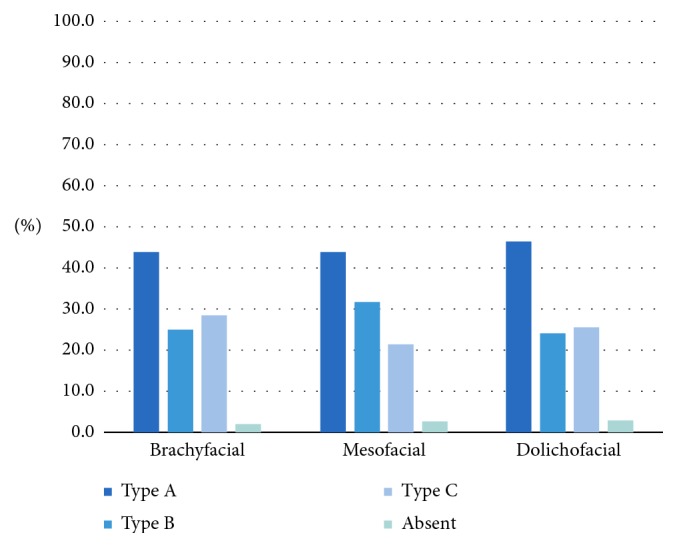
Association between the type of loop (regardless of side) and the facial type.

The anterior loop type overall was not significantly associated with the facial type (*p* > 0.05; Figure 10), and Type A was the most prevalent type overall.

## 4. Discussion

The present study demonstrated a significant association between the various facial types and anatomical variations of the mandibular canal. Brachyfacial and mesofacial types mostly had canal paths running close to the root apexes (65.4% (*n*=15) and 55.4% (*n*=56) of the studied canals, respectively). In dolichofacial types, the main canal mostly ran closest to the base of the jaw, with branches extending to the apexes (68.2% (*n*=45) of the canals) and only 4.5% (*n*=3) of cases showed a canal running near the root tips. Mesofacial types had a lower prevalence of bifid mandibular canals than the other facial types.

Our results contrast with those presented by Schmidt et al. [6], who found no significant association between mandibular canal variants and facial types. This may be because they used panoramic radiographs for their examinations.

Rossi et al. [11] reported that genetic variation and ethnicity seemed to influence anatomical variations of the mandibular canal, with the prevalence of the types varying with geographic location. Although the current study did not set out to select samples by race and region, our sample contained a higher frequency of Caucasians, Africans and mulattos, and fewer Indian and Asian individuals; the current study results corroborated the finding of significant association between different facial types and different facial morphology of the mandibular canal.

CBCT has been shown to be a reliable tool to identify and measure the anterior loop [12]. Uchida et al. [13] found differences smaller than 0.1 mm between the anatomic measurements of the anterior loop in CBCT images, confirming the reliability of the CBCT for this purpose. Some studies have also used CBCT to measure the length and diameter of the anterior loop and incisor canal [13–15], while others have reported the prevalence of the incisive canal in certain populations [12, 16, 17]. Chen et al. [14] found racial influences when comparing the anterior loop measurement and reported that the anterior loop is longer in Taiwanese (−1.81 + 7.61 mm) than in American individuals (6.22 ± 1.81 mm).

In this study, we assessed anterior loop variants on CBCT images (Types A, B, and C), as previously described by Li et al. [17] and Do Nascimento et al. [18]. We found a higher prevalence of the anterior loop (Type A) and the lowest prevalence of Type B.

In terms of bifid canals, irrespective of the side studied, the Type 1 canal was the most frequent, accounting for about 12% of cases, and there was no significant difference in the prevalence between the sexes and with age, which is in agreement with results reported by Li et al. [17] in a Chinese population and differs from those of Fu et al. [19], who studied Taiwanese individuals, in which the prevalence of the bifid canal was higher in males.

This research is clinically relevant because knowledge about the correct location and anatomical variations of the mandibular canal is essential for the success of numerous dental procedures involving the jaw, for instance, in anesthesia in routine dentistry practice, endodontics, periodontics, and pediatric dentistry, as well as for more invasive interventions, such as orthognathic surgery and implant installation [10, 20–23].

Stella and Tharanon [24] reported that mandibular canal anatomy may vary according to a number of factors, such as age, sex, race, and development of the alveolar bone. The results presented here contradicted their statement, as no significant associations of facial canal morphology and anatomical variations were found with age and sex. However, important factors identified in this study suggest that facial type may be considered an indicator of the location and morphology of the mandibular canal in clinical situations, which is important for planning surgical and dental procedures that require optimizing the quality of anesthesia.

Considering the importance of the issue and the lack of similar studies on the association between facial type and anatomical changes in the mandibular canal based on CBCT, further research is needed to verify the methodological approach used here to clarify the influence of facial types on the localization and morphology of the mandibular canal.

## 5. Conclusion

The morphology of the mandibular canal and its variations present significant association with different facial types, regardless of age, sex, or the side of the face studied.

## Figures and Tables

**Table 1:** Absolute (*n*) and relative (%) frequency of the association between the prevalence of anatomical variations of the mandibular canal on the right side and facial type.

Variable		Total		Facial type						*p* value
				Brachyfacial		Mesofacial		Dolichofacial		
		*n*	%	*n*	%	*n*	%	*N*	%	
Location: right side	1	86	49.4	33	63.5	52	58.4	1	3.0	<0.0001
	2	56	32.2	7	13.5	26	29.2	23	69.7	
	3	32	18.4	12	23.1	11	12.4	9	27.3	

Bifid type, right side	I	18	10.3	10	19.2	4	4.5	4	12.1	0.0258
	II	11	6.3	2	3.8	5	5.6	4	12.1	
	III	2	1.1	0	0.0	2	2.2	0	0.0	
	IV	1	0.6	0	0.0	0	0.0	1	3.0	
	Absent	142	81.6	40	76.9	78	87.6	24	72.7	

Anterior loop, right side	A	77	44.3	22	42.3	34	38.2	21	63.6	0.0088
	B	53	30.5	14	26.9	36	40.4	3	9.1	
	C	43	24.7	15	28.8	19	21.3	9	27.3	
	Absent	1	0.6	1	1.9	0	0.0	0	0.0	

**Table 2:** Analysis of the association between the prevalence of anatomical variations of the mandibular canal on the left side and facial type.

Variable		Total	Brachyfacial	Mesofacial	Dolichofacial	*p* value
		*n* (%)	*n* (%)	*n* (%)	*n* (%)	
Location: left side	1	83 (48.0)	35 (67.3)	46 (52.3)	2 (6.1)	<0.0001
	2	60 (34.7)	8 (15.4)	30 (34.1)	22 (66.7)	
	3	30 (17.3)	9 (17.3)	12 (13.6)	9 (27.3)	

Bifid type, left side	I	20 (11.6)	8 (15.4)	8 (9.1)	4 (12.1)	0.1009
	II	8 (4.6)	1 (1.9)	2 (2.3)	5 (15.2)	
	III	1 (0.6)	0 (0.0)	1 (1.1)	0 (0.0)	
	IV	1 (0.6)	0 (0.0)	1 (1.1)	0 (0.0)	
	Absent	143 (82.7)	43 (82.7)	76 (86.4)	24 (72.7)	

Anterior loop, left side	A	78 (45.1)	24 (46.2)	44 (50.0)	10 (30.3)	0.3295
	B	45 (26.0)	12 (23.1)	20 (22.7)	13 (39.4)	
	C	42 (24.3)	15 (28.8)	19 (21.6)	8 (24.2)	
	Absent	8 (4.6)	1 (1.9)	5 (5.7)	2 (6.1)	

**Table 3:** Analysis of the association between the prevalence of anatomical variations of the mandibular canal and facial type (independent of side).

Variable		Facial type				*p* value
		Total	Brachyfacial	Mesofacial	Dolichofacial	
		*n* (%)	*n* (%)	*n* (%)	*n* (%)	
Location: independent of side	1	169 (48.7)	68 (65.4)	98 (55.4)	3 (4.5)	<0.0001
	2	116 (33.4)	15 (14.4)	56 (31.6)	45 (68.2)	
	3	62 (17.9)	21 (20.2)	23 (13.0)	18 (27.3)	

Bifid type: independent of side	I	38 (11.0)	18 (17.3)	12 (6.8)	8 (12.1)	0.0032
	II	19 (5.5)	3 (2.9)	7 (4.0)	9 (13.6)	
	III.	3 (0.9)	0 (0.0)	3 (1.7)	0 (0.0)	
	IV	2 (0.6)	0 (0.0)	1 (0.6)	1 (1.5)	
	Absent	285 (82.1)	83 (79.8)	154 (87.0)	48 (72.7)	

Anterior loop type: independent of side	A	155 (44.7)	46 (44.2)	78 (44.1)	31 (47.0)	0.7553
	B	98 (28.2)	26 (25.0)	56 (31.6)	16 (24.2)	
	C	85 (24.5)	30 (28.8)	38 (21.5)	17 (25.8)	
	Absent	9 (2.6)	2 (1.9)	5 (2.8)	2 (3.0)	

## Data Availability

The data used to support the findings of this study are available from the corresponding author upon request.
